# Sodium glucose co-transporter 2 inhibitor-associated euglycaemic diabetic ketoacidosis in the emergency peri-operative period: a systematic review

**DOI:** 10.1007/s00540-025-03570-2

**Published:** 2025-08-31

**Authors:** Dennis Perez Castillo, Leanne Hall, Siva Senthuran, Elliot Fox, Sananta Dash, Clare Heal

**Affiliations:** 1 James Cook University, Townsville, Australia; 2 James Cook University, Mackay, Australia; 3 Townsville University Hospital, Townsville, Australia

**Keywords:** Sodium-glucose co-transporter 2 inhibitors (SGLT2i), Euglycaemic diabetic ketoacidosis (EuDKA), Emergency surgery, Perioperative complications, Type 2 diabetes mellitus

## Abstract

Perioperative euglycaemic diabetic ketoacidosis (EuDKA) is a rare but life-threatening complication associated with sodium-glucose co-transporter-2 inhibitors (SGLT2i). It is characterised by ketonaemia, acidosis, and normal serum glucose. Whilst guidelines advise withholding SGLT2i prior to elective surgery, limited guidance exists for emergency procedures. This systematic review aimed to describe EuDKA cases following emergency surgery, identify patient characteristics, and examine contributing risk factors. A search of electronic databases up to April 2024 identified 30 cases from 21 publications. In most cases, EuDKA onset occurred within three days postoperatively (range: intraoperative to 10 days). Reported risk factors included inadequate SGLT2i withholding time, poor glycaemic control, morbid obesity, major surgery, intercurrent illness, suboptimal intraoperative diabetes management, and delayed gastrointestinal absorption. Morbidity was significant: ten patients required intensive care, two required intubation and ventilation, two received dialysis, and one underwent exploratory laparotomy. No deaths were reported. Due to atypical biochemical findings and non-specific symptoms, EuDKA remains under-recognised. Clinicians are advised to maintain a high index of suspicion, ensure appropriate perioperative insulin management, conduct vigilant laboratory monitoring, and provide patient education to reduce the risk in emergency surgical settings.

**Supplementary Information:**

The online version contains supplementary material available at 10.1007/s00540-025-03570-2.

## Background

Type 2 diabetes (T2DM) impacts almost 1.3 million Australians, with this number continuing to rise as a result of the current obesity epidemic and an ever-ageing population [1]. Many of these individuals live with a number of co-morbidities including chronic kidney disease and cardiovascular disease, significantly affecting their quality of life. As a result, T2DM is a burgeoning global issue with a growing need for effective and reliable treatment options. In response to this growing demand, a new class of glucose-lowering medication, the sodium-glucose co-transporter-2 inhibitor (SGLT2i), was made available in Australia in 2013 [2]. Their novel mechanism of action involves the inhibition of renal glucose absorption, promoting glycosuria and improved glucose control, and is associated with a reduction of HbA1c by up to 0.9% compared to placebo [3–5]. In addition to its glycaemic effects, SGLT2i display a number of benefits which include weight loss, blood pressure reduction, and renal-protective properties [4, 6].

Currently, only dapagliflozin and empagliflozin, alongside their combinations with other glucose-lowering medications, are available in Australia [7]. The indications for this class have expanded from 2nd line management of T2DM to include chronic kidney disease and heart failure as of 2022 [8]. The therapeutic value of this medication is evident as reflected with the spike in Pharmaceutical Benefit Scheme (PBS) prescriptions of SGLT2i from 750,000 prescriptions in 2016 to greater than 2.3 million in 2019 [9], and it is estimated that as of 2022, over 250,000 Australians are on an SGLT2i [10]. In light of the wide clinical uptake of SGLT2i’s, it is imperative to judge their safety profile. In May 2015, the US Food and Drug Administration (FDA) released a statement outlining the risk of euglycaemic diabetic ketoacidosis (EuDKA) associated with this medication, causing widespread concern [11].

SGLT2i-induced-EuDKA is a rare, yet life-threatening adverse event defined by ketonaemia (total serum ketones ≥ 0.6 mmol/L or serum β-hydroxybutyrate ≥ 0.5 mmol/L), acidosis (pH ≤ 7.30, serum bicarbonate ≤ 18 mmol/L or anion gap (AG) > 10) in the context of normal serum glucose levels (< 13.9 mmol/L) [12, 13]. The event rate of SGLT2i-induced-EuDKA is estimated to be between 1.3 and 4.9 cases per 1000 patient years [14, 15]. SGLT2 inhibitors predispose to EuDKA by promoting urinary excretion of sodium and glucose independently of insulin. This mechanism reduces circulating insulin levels and stimulates glucagon secretion, thereby enhancing lipolysis and ketogenesis. In addition, the associated osmotic diuresis contributes to a negative fluid balance, which may further exacerbate the risk of ketoacidosis [12]. Known risk factors include intercurrent illness, missed insulin, and reduced caloric intake, yet perhaps most notably is the risk of EuDKA in the perioperative setting [13, 16]. An approved package insert change by the US FDA, amongst worldwide alerts such as the 2022 alert update for perioperative SGLT2i usage in Australia, saw a new recommendation to withhold SGLT2i for a minimum of 3 days prior to surgery to reduce the risk of EuDKA [17, 18]. Preliminary studies have sought to evaluate the efficacy of these recommendations; however, it has been highlighted that in the case of emergency procedures it is often impossible to withhold the offending agent, resulting in the ongoing occurrence of EuDKA [19].

An initial search of MEDLINE, Open Science Framework, the Cochrane Database of Systematic Reviews, and JBI Evidence Synthesis was performed, and no current or underway systematic or scoping reviews on the topic of emergency peri-procedural SGLT2i-induced EuDKA were identified. We conducted a systematic review of case reports and case series of SGLT2i-induced EuDKA in emergency perioperative populations up until April 2024 with two primary objectives: Firstly, to describe the demographic, clinical, and biochemical characteristics of patients with T2DM who develop EuDKA in the immediate postoperative period after undergoing emergency surgery and receiving SGLT-2 inhibitors. Secondly, to identify and analyse the risk factors that contribute to increased morbidity and mortality in patients with T2DM who develop EuDKA following emergency surgery.

## Methods

### Search strategy

A systematic search of MEDLINE, Scopus, CINAHL, EMCARE, PubMed, and Web of Science databases was conducted on April 7, 2024, to identify relevant studies that identified cases of patients who underwent emergency procedures and subsequently developed euglycaemic diabetic ketoacidosis (EuDKA) in the perioperative period. A combination of the following four major search terms and their derivatives was used for all databases: “type 2 diabetes mellitus” “emergency surgery” “SGLT-2 inhibitor” and “euglycaemic diabetic ketoacidosis” No restrictions were placed on language or date. The full search strategy is provided in Supplementary Table 1. References of review articles and included studies were searched to identify any articles not identified in the initial search. The study protocol was registered with PROSPERO (CRD42024551884) prior to conducting formal database searching.

### Eligibility criteria

This study was conducted in accordance with the Preferred Reporting Items for Systematic Reviews and Meta-Analysis (PRISMA) guidelines [20]. Following the removal of duplicate articles, two authors D.P.C. and E.F. independently screened the titles and abstracts to assess relevance for review and collected full-text articles which were also assessed for final inclusion into the study. Any disagreements were resolved through discussion. The criteria for inclusion were primary studies that reported patients over the age of 18 years diagnosed with T2DM receiving SGLT-2 inhibitors perioperatively who underwent an emergency procedure and did not cease SGLT-2 usage more than 48 h prior to surgery. The cases must then have stated the development of EUDKA (defined as pH < 7.3, anion gap [AG] > 10, serum glucose < 13.9 mmol/L and blood/urine ketone positivity) intra or postoperatively up to 30 days. Review articles, studies including patients undergoing elective procedures, those who ceased SGLT-2i usage > 48 h prior to a procedure, patients not fitting the EuDKA criteria, pregnant patients, and studies from which relevant data could not be extracted were excluded. Inclusion and exclusion criteria are summarised in Supplementary Table 2.

### Quality appraisal

The methodological quality of the included studies was assessed using the Joanna Briggs Institute (JBI) Critical Appraisal Tools: checklists for case reports and case series were utilised for the respective study design and are provided in Supplementary Tables 3 and 4. Studies were assessed for quality by two independent reviewers (D.P.C. and E.F.), with disagreements resolved through mutual discussion.

### Data extraction

Data were extracted by D.P.C. and reviewed by E.F. into an Excel spreadsheet and included study design, country and year of publication, baseline patient demographics including age, sex, body mass index (BMI), co-morbidities, SGLT2i type, oral hypoglycaemic agents and other medications, type of surgery, length of time SGLT2i were ceased (if ceased), time to onset of EuDKA, perioperative management of diabetes, anaesthesia, trigger to identification/symptoms of EuDKA, biochemical parameters at time of EuDKA diagnosis (including serum glucose, HbA1c, pH, serum bicarbonate [HCO3], anion gap [AG], partial pressure of arterial carbon dioxide [PaCO2] serum and/or urine ketones and glycosuria), management of EuDKA, complications and outcomes.

### Analysis

Descriptive statistics were used to report and summarise quantitative data, with continuous data presented as mean (standard deviation (SD)) or median (range), and categorical data as percentage values. No attempts were made to meta-analyse data due to the clinical heterogeneity of articles included.

## Results

### Publication screening

**Fig. 1:**
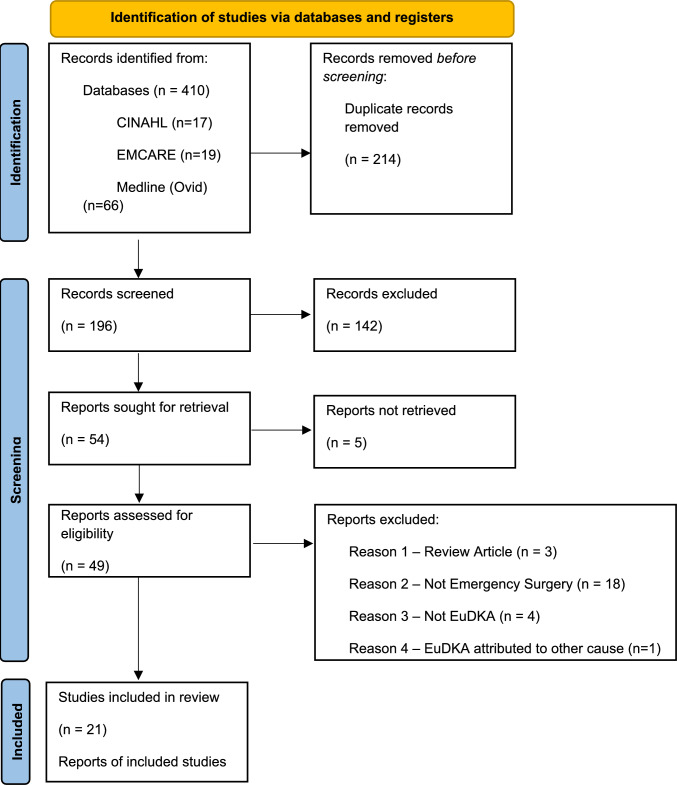
PRSIMA Flowchart

The search strategy identified 410 citations. After removal of 214 duplicates, 196 articles were screened via title and abstract, with 49 reports assessed in full text, culminating in 21 studies, with 30 cases, being included in the final review (Fig. 1.) [21–41]. The quality of all included articles was adequate as per the JBI appraisal process.

All articles included involved the reporting of a single or series of cases. The majority of cases were from the USA (*n* = 6), followed by Japan (*n* = 4), Taiwan (*n* = 3) and Australia (*n* = 2). The reports were all published between 2016 and 2023, with more than half (*n* = 12) published after 2019. Details of individual case reports are provided in Table 1.

**Table 1:** Basic Characteristics and Outcomes of Individual Case Reports

Author/Year	Age/Gender	BMI	HbA1c	SGLT2i Cessation time	Surgery	Hospital outcome
Abu-Amer N et al. 2019 [21]	51y, F	N/A	N/A	24–48 h	Laparoscopic gastric wedge resection for gastric perforation	Complicated (Additional surgery)
Bteich F et al. 2019 [22]	58y, F	N/A	N/A	24–48 h	VP Shunt Exchange	Complicated (ICU)
Chandrakumar HP et al. 2021 [23]	68y, F	N/A	12.8	Not Ceased	Trabeculectomy for Acute Angle Glaucoma	Not Complicated
Fisker F et al. 2019 [24]	51y, M	28	7.5	< 24 h	Local debridement of infected diabetic foot + incision and drainage of plantar abscess	Not Complicated
Fustiga J et al. 2022 [25]	77y, F	N/A	7.8	Not Ceased	Laparoscopic cholecystectomy	Not Complicated
Horikoshi R et al. 2019 [26]	Case 1: 48y, F	N/A	N/A	Not Ceased	CABG	Not Complicated
	Case 2: 53y, M	N/A	N/A	Not Ceased	CABG	Not Complicated
Hung KC et al. 2021 [27]	50y, M	24.9	N/A	Not Ceased	ORIF of right femoral neck fracture, then further procedures for left acetabulum and right tibia fractures	Not Complicated
Ito T et al. 2022 [28]	55y, M	N/A	8	< 24 h	Emergency off-pump coronary artery bypass grafting (OPCAB)	Not Complicated
Jhaveri U et al. 2019 [29]	41y, F	41	11.7	< 24 h	Incision and drainage of abscess	Not Complicated
Kitahara C et al. 2021 [30]	59y, M	24	9.4	24–48 h	Thoracoscopic debridement and intrathoracic lavage	Not Complicated
Leon CG et al. 2020 [31]	42y, F	> 40	N/A	< 24 h	Total hysterectomy	Complicated (ICU and Dialysis)
Lindsay PJ et al. 2020 [32]	51y, M	> 40	9	Not Ceased	Debridement for Fournier's gangrene	Complicated (ICU)
Mehta PB et al. 2022 [33]	Case 1: 65y, M	N/A	N/A	Not Ceased	Cerebral angiography	Not Complicated
	Case 2: 80y, M	N/A	N/A	Not Ceased	Cystoscopy	Not Complicated
	Case 3: 50y, M	N/A	N/A	Not Ceased	Catheter-directed thrombolysis of R popliteal graft	Not Complicated
	Case 4: 80y, M	N/A	N/A	Not Ceased	Biventricular ICD placement	Not Complicated
	Case 5: 71y, M	N/A	N/A	Not Ceased	C4-C5 laminectomy	Not Complicated
Ritchie DT et al. 2023 [34]	60y, M	N/A	11.6	< 24 h	Total hip replacement	Complicated (ICU)
Takemoto Y et al. 2019 [35]	71y, F	24.8	6	< 24 h	Laparoscopic cholecystectomy	Not Complicated
Tsai MK et al. 2019 [36]	Case 1: 60y, F	N/A	12	Unclear	ORIF for tibia fracture (day 1) + spinal fusion (day 9)	Complicated (ICU)
	Case 2: 63y, F	N/A	11	Unclear	ORIF for humerus and femur fracture	Complicated (ICU and Ventilation)
Ullah S et al. 2016 [37]	40y, F	N/A	N/A	Not Ceased	Incision and drainage for cellulitis	Complicated (ICU)
Wang Q et al. 2022 [38]	57y, F	N/A	9.3	< 24 h	Laparoscopic distal pancreatectomy and emergency laparotomy 9 days following first operation	Complicated (ICU and Emergency Laparotomy)
Wang R et al. 2021 [39]	Case 1: N/A	N/A	N/A	< 24 h	CABG	Not Complicated
	Case 2: N/A	N/A	N/A	< 24 h	Laminectomy	Not Complicated
	Case 3: N/A	N/A	N/A	24–48 h	Craniotomy	Not Complicated
	Case 4: N/A	N/A	N/A	24–48 h	Laparotomy and bowel resection	Not Complicated
Wong YC et al. 2021 [40]	57y, F	N/A	N/A	< 24 h	ORIF for proximal femoral shaft fracture	Complicated (ICU, Ventilation and Dialysis, Amputation)
Yeoh HL et al. 2021 [41]	61, M	N/A	10	< 24 h	Percutaneous pedicle screw fixation of spinal fractures and ORIF of left ulna shaft fracture	Not Complicated

*SGLT2i* sodium glucose co-transporter 2 inhibitor, *VP* ventriculoperitoneal, *CABG* coronary artery bypass grafting, *ORIF* open reduction internal fixation, *ICD* implantable cardioverter-defibrillator*, ICU* intensive care unit

### Case characteristics

There were 30 individual cases of SGLT2i-induced EuDKA, all of which were confirmed T2DM. The median age was 57.5 years (range: 40–80), 13 cases were male, 13 female, and the sex was not recorded in four cases. BMI was reported in 7/30 (23.3%) cases, with a median BMI of 28 kg/m^2^ (range: 24–41), which is in the overweight range. Of these seven cases, three (43%) cases reported morbid obesity (BMI > 40 kg/m^2^). Biochemical and clinical outcomes were worse in these morbidly obese cases: 2/3 had severe acidosis (pH < 7, HCO3 < 10 mmol/L), 2/3 required ICU admission, and 1/3 needed haemodialysis for refractory acidosis. Baseline HbA1c was reported in 13/30 (43.3%) cases, with a mean of 9.7% (SD ± 2%). Only 1/13 (8%) cases had HbA1c below 7%. The HbA1c was > 10% in 6/13 (46%) cases. In this high HbA1c group, severe acidosis occurred in 4/6 (67%), in which 3/6 (50%) required ICU admission and 1/6 (17%) needed intubation and ventilation.

The most commonly reported co-morbidities were hypertension (*n* = 8), coronary artery disease (*n* = 6), morbid obesity (*n* = 3), obstructive sleep apnoea (*n* = 2) and cancer (*n* = 2). Co-morbidities were not reported in 9/30 (30%) cases. Co-morbid cardiovascular disease was not associated with adverse outcomes in our case cohort. The SGLT2i prescribed was reported in 26/30 cases: empagliflozin (*n* = 15), dapagliflozin (*n* = 6), canagliflozin (*n* = 4) and ertugliflozin (*n* = 1). Diabetic medication regimes for 20/30 cases were recorded, with the most common regimen of SGLT2i + metformin seen in 6/20 (30%) cases. Insulin was prescribed alongside an SGLT2i as part of routine management in 4/20 (20%) cases. One case involved the use of five different hypoglycaemic agents at one time.

### Surgical characteristics

Reported emergency surgeries for each case are described in detail in Supplementary Table 5; however, the most commonly reported surgical specialty was orthopaedics (*n* = 8), with open reduction and internal fixation (ORIF) of trauma fractures being the most common surgery type (*n* = 5). Other common surgery types reported were coronary artery bypass grafting (CABG) (*n* = 4) and soft tissue surgeries (Incision and drainage, debridement) (*n* = 4). Of the eight orthopaedic cases, 2/8 (25%) had severe acidosis (pH < 7) compared to 1/22 (5%) of non-orthopaedic cases, and 5/8 (63%) had significant metabolic derangement (HCO3 < 10 mmol/L). Worse clinical outcomes were observed, with 4/8 (50%) requiring ICU admission and 2/8 (25%) needing intubation, ventilation, or dialysis.

Anaesthetic technique was only described in five studies in which general anaesthesia was utilised. The perioperative fasting period was also only reported in a small number of cases (n = 7). The shortest fasting period was five hours, and the longest was two days.

### Perioperative management of SGLT2i

Preoperative SGLT2i management was reported in 28/30 cases. Of these, SGLT2i was not ceased in 12/28 (42.9%), ceased for less than 24 h in 11/28 (39.3%) or ceased between 24 and 48 h in 5/28 (17.9%) cases. One case reported SGLT2i commenced on the day prior to the procedure, initiated by the inpatient team. Cases where SGLT2i was not withheld had worse biochemical parameters at diagnosis, with a median pH of 7.19 vs 7.23 and median HCO3 of 11.8 mmol/L vs 15.4 mmol/L. The risk of ICU admission, however, did not seem to be overtly related to the withholding period of a SGLT2i. ICU admission was required in 2/12 cases (17%) where the SGLT2i was not withheld, compared to 5/16 cases (31%) where the SGLT2i was withheld.

### Perioperative management of diabetes

Perioperative management of diabetes prior to the onset of EuDKA was described in 12/30 (40%) cases, with most involving a regimen of pre or intra-operative insulin infusion with fluids and glucose (*n* = 8), whilst only fluids were commenced in four cases. Intra-operative diabetes protocols were not mentioned in 18 cases. Pre-operative ketone and acidosis screening was performed in 4/30 (13%) and 2/30 (6%) cases respectively. In patients who were co-prescribed insulin with an SGLT2i, EuDKA was less severe, with 4/4 (100%) experiencing milder metabolic acidosis (pH > 7 and HCO_3_ > 10) and 1/4 (25%) requiring ICU admission.

### Euglycaemic DKA characteristics

The time to diagnosis of EuDKA was reported for 21/30 (70%) cases. Of these, EuDKA was diagnosed intra-operatively in 4/21 (19%) patients, postoperatively but within the same day of the procedure in 7/21 (33.3%) patients, and 3/21 (14.3%) patients in each of the first and second postoperative days, respectively. The longest reported period between surgery and diagnosis of EuDKA was 10 days. EuDKA was identified through laboratory data in 15/30 (50%) cases. Reasons for investigation were described in 10 cases: nine due to abnormal routine tests and one due to EuDKA as an initial differential. Symptoms as a means for identification were only reported in 10/30 (33.3%) of cases, with most being largely unspecific, e.g., nausea and/or vomiting, dyspnoea, tachycardia or a reduction in appetite. The reason for EuDKA identification was not reported in 5/30 (16.7%) cases.

With respect to the biochemical parameters used to diagnose EuDKA, ketone positivity was evident in all but one case. Ketone positivity via solely blood samples was reported in 16/29 (55%) cases, 3/29 (10%) via urine and 10/29 (35%) used both. Blood ketosis was identified via total serum ketones in 12/26 (46%) cases, and β-hydroxybutyrate (BHB) in 14/26 (54%) cases. Urine ketones were either reported at levels of positivity (i.e. 3 +) or at specific values, complicating analysis. Blood gas analysis was used in every case, showing a median pH of 7.19 (range 6.94–7.36). HCO3, AG and PaCO2 values were also recorded in 27 (90%), 15 (50%) and 18 (60%) cases respectively. The median and ranges for each respective variable are reported in Table 2. Blood glucose levels were reported in all 30 cases; however, four of these were reported as a range rather than discrete values. For the remaining 26 cases, the median serum glucose level was 9.98 mmol/L (range: 6.1–17.5).

**Table 2:** Characteristics of SGLT2i-EuDKA secondary to Emergency Surgery

Category	Details	Results
Demographics	Age (*n* = 26), mean ± SD	58.4 ± 10.96
	Gender (*n* = 26)	Male (*n* = 13), Female (*n* = 13),
	BMI (*n* = 7), median [range]	28 [24-40]
Time to Diagnosis	Intraoperative	4/30 (13.3%)
	Day of Procedure	7/30 (23.3%)
	POD1	3/30 (10%)
	POD2	3/30 (10%)
	POD 3 +	4/30 (13.3%)
	Not reported	9/30 (30%)
Precipitating Factors	Surgical Stress	15
	Inadequate Caloric Intake	10
	Infection	6
	Inadequate Intraoperative Insulin	3
	Hypovolaemia	2
	Misdiagnosis Delaying Treatment	2
	Poorly Controlled Diabetes	1
	Not Reported	9
Trigger for Identification	Laboratory Data	15/30 (50%)
	Symptomatic	10/30 (33.3%)
	Not Reported	5/30 (16.7%)
Blood Gas Analysis, median [range]	pH (*n* = 30)	7.19 [6.94–7.36]
	HCO_3_, mmol/L (*n* = 27)	12.0 [2.0–28.0]
	AG, mmol/L (*n* = 15)	20.8 [12.0–36.2]
	pCO_2_, mmHg (*n* = 18)	33.7 [5.0–56.7]
Plasma Ketones, median [range]	Total Ketones, mmol/L (*n* = 10)^*^	7.3 [3.8–12.4]
	BHB, mmol/L (*n* = 14)^**^	5.0 [1.1–10.9]
Plasma Glucose, median [range]	Random Blood Glucose, mmol/L (*n* = 30)^***^	9.1 [6.1–17.5]
	HbA1c (*n*= 13)	9.4 [6–12.8]

*POD* post-operative day, *BHB* b-hydroxybutyrate, *SGLT2i* sodium-glucose co-transporter 2 inhibitors

*Normal range: less than 0.6 mmol/L

**normal range: less than 0.5 mmol/L

***normal range: less than 13.9 mmol/L

Most cases attributed the development of EuDKA to one or more precipitating factors, of which the most frequently identified were surgical stress (*n* = 15), reduced caloric intake (*n* = 10), infection (*n* = 6) and inadequate intraoperative insulin (*n* = 3).

### Treatment of EuDKA

Treatment details were collected from 24/30 cases, in which fluids (*n* = 24), glucose (*n* = 24), insulin (*n* = 22) and occasionally bicarbonate (*n* = 4) and electrolyte (n = 6) administration were used. Several cases described further management, with 10/21 (47%) requiring ICU admission, two needing intubation and ventilation, and two receiving dialysis. Misdiagnosis was a common theme, with two patients receiving unnecessary explorative surgeries and another receiving vasopressor support, which resulted in dry gangrene of toes and subsequent amputation.

### Outcomes of EuDKA

Patient outcomes were only reported in 21/30 (70%) cases, in which all made a full recovery from ketoacidosis. Time to recovery ranged from eight hours to six days. 8/21 (38%) cases reported permanent discontinuation of SGLT2i on discharge. Whether therapy was resumed after discharge is unknown. There were no reported deaths.

## Discussion

In this systematic review of case reports, we identified 30 unique patient experiences from 21 primary studies describing the course of perioperative SGLT2i-induced EuDKA published between 2016 and 2023.

### Patient characteristics

An investigation of pre-morbid characteristics identified a number of possible precipitating factors.

The median BMI amongst the small subset of reported cases (*n* = 7) was 28 kg/m^2^; with three patients reported as morbidly obese (BMI > 40 kg/m^2^), all of whom experienced complicated clinical courses. Whilst obesity is a recognised risk factor for operative and anaesthetic complications, and previous studies have linked morbid obesity to worse outcomes in DKA, the limited case number in this review precludes definitive conclusions [42, 43]. This observation provides an opportunity for further research to elucidate the relationship between obesity and SGLT2i-EuDKA outcomes. Nonetheless, clinicians may consider heightened vigilance in patients with morbid obesity until this hypothesis is verified.

Baseline HbA1c was reported in only 13 of 30 cases (43%), severely limiting the strength of any subgroup analysis. Treatment targets for T2DM suggest a target HbA1c of < 7% for adequate glycaemic control [44]. Amongst the 13 reported cases, 12 exceeded this threshold, and six had markedly elevated HbA1c values (> 10%). This threshold was selected to dichotomise this cohort at a HbA1c of 10% because of an association with poorer DKA outcomes at this level in the literature [45]. Within this high-HbA1c subgroup, more severe acidosis and higher rates of ICU admission were observed. Conversely, the only case with a well-controlled HbA1c (6.0%) experienced a relatively mild course of EuDKA [35]. Existing literature suggests elevated HbA1c increases ketosis risk due to lower effective circulating insulin and elevated counter-regulatory hormones like glucagon, cortisol, and catecholamines, promoting lipolysis and ketogenesis [46]. SGLT2i further compounds this effect via its glucagon stimulating effects [47]. The alert update by the Diabetes Society and Australian New Zealand College of Anaesthetists mentions that a high HbA1c (> 9%) is to be a risk factor for developing DKA in patients on an SGLT2i [18]. Whilst these findings raise the possibility that poor long-term glycaemic control may predispose to more severe perioperative EuDKA, the small sample size and incomplete reporting prevent conclusive interpretation. The observed association should therefore be interpreted as hypothesis-generating only.

### SGLT2i management

We observed that empagliflozin was the most frequently implicated SGLT2i in our case cohort. Ata et al. suggest that canagliflozin is most commonly associated with EuDKA; however, this is likely the result of canagliflozin being the earliest developed drug in this class [48]. Our findings reflect recent prescribing trends, with empagliflozin being the most commonly prescribed in its class, with 1.4 million PBS prescriptions in 2022–23, presumably the result of its significant cardiovascular benefits and reduced availability of canagliflozin in Australia [49–51]

Emergency procedures pose a challenge for clinicians attempting to hold SGLT2i’s. The average half-life for SGLT2i’s ranges from 11 to 13 h, with almost five half-lives (2.5 days) for 97% of the drug to be eliminated through the kidneys [52]. Consensus guidelines recommend the cessation of these drugs two to three days prior to surgery where possible; however, some sources suggest longer withdrawal periods of five to seven days before major surgeries, suggesting there is room for further refinement of guidelines [18, 53]. Nonetheless, a major risk of emergency procedures is the inability to adequately withhold the SGLT2i, contributing to poor EuDKA outcomes [54]. We described 16 cases where an SGLT2i was ceased at most for 48 h, and these cases were associated with milder disease and better outcomes.

Additionally, we identified 5/30 (16.7%) cases which described a period of acute illness and reduced oral intake prior to surgery [21, 25, 30, 32, 35]. This included one case described by Fustiga et al. who continued to take her empagliflozin despite three days of significant vomiting and abdominal pain [25]. Consequently, we investigated the role of sick day precautions in the literature. National prescribing guidelines suggest providing all new SGLT2i users with a written sick day management plan [44, 55, 56]. A review of sick day management recommendations by Watson et al. concluded that the temporary cessation of the SGLT2i, home capillary ketone monitoring, and utilisation of insulin was recommended on sick days to avoid the risk of ketoacidosis [57]. Regarding our patient cohort, there were no instances of sick day precautions being made to prevent EuDKA. Based on existing evidence, we suggest that such precautions may have reduced the severity or prevented the development of EuDKA in cases of acute illness, and as such recommend the provision of clear, written sick day precautions for all SGLT2i users.

### Operative considerations

Orthopaedic procedures were the most frequently reported surgical specialty in this review; however, the cases included within this group varied widely in nature, ranging from ORIF of traumatic fractures to total hip replacements. Given this heterogeneity in surgical type, trauma severity, and perioperative physiological stress, no reliable conclusions can be drawn about a causal association between orthopaedic surgery and increased EuDKA severity. It is likely this observation reflects reporting bias as ORIFs may be over-represented in emergency procedures performed. Although a greater proportion of severe outcomes were observed in this subgroup, these findings are likely confounded by factors such as surgical complexity, pre-existing comorbidities, and the acute physiological insult associated with trauma-related admissions. As such, the apparent association should be interpreted with caution, and as a result, we suggest a specialty-specific review.

One case by Hung et al. offered an additional perspective by describing the use of Surgical Plethysmographic Index (SPI) monitoring during an ORIF procedure [27]. SPI-guided analgesia aims to quantify physiological stress and optimise analgesic delivery and has been associated with improved perioperative outcomes [58]. Invasive surgical procedures induce significant physiological stress that may trigger a hypercatabolic state, potentially contributing to the development of EuDKA [59–61]. Whilst this case raises an interesting hypothesis, it is based on a single patient and should be interpreted with caution. At present, there is no evidence to support the use of SPI monitoring as a preventative strategy for EuDKA and its utility in this context remains unproven. Further research on its impact in this context is warranted.

Evidence indicates improper fluid and glucose management, as well as anaesthetic technique, can affect perioperative glucose in diabetic patients, increasing the risk of EuDKA. [62, 63] Unfortunately, these points were rarely reported in our case cohort. Nevertheless, three cases identified early cessation of intra-operative insulin as a predisposing factor to EuDKA [28, 31, 32]. Consensus regarding optimal peri-operative diabetes management is growing, with case reports and series reporting similar findings that insulin administration pre-operatively may be protective against the development of EuDKA [33, 64]. Ito et al. suggest initiating continuous insulin infusion (CII) and glucose administration prior to the initiation of surgery and continuing until oral intake is sufficient may be beneficial in reducing the risk of ketoacidosis [28].

Preoperative screening for ketosis and acidosis in patients taking SGLT2 inhibitors, particularly those undergoing emergency procedures, represents a potentially valuable risk mitigation strategy. Existing guidelines recommend perioperative ketone assessment in patients who are unwell or otherwise at elevated risk [65]. However, in this review, preoperative ketone testing was documented in only four cases, and acid–base assessment in just two, underscoring a gap in routine evaluation or reporting practices. Although the current evidence base is limited, incorporating targeted metabolic screening into perioperative protocols may facilitate earlier recognition and prevention of EuDKA. Further research is warranted to establish its clinical utility.

### Diagnosis of EuDKA

We report trends regarding time to EuDKA to onset in 21/30 (70%) cases. Of these, one-third were diagnosed intra-operatively, and over 80% occurred before POD3. Interestingly, four cases described delayed EuDKA onset, ranging from five to ten days post-surgery [21, 23, 36, 38]. We investigated the pharmacokinetics of SGLT2i by collecting glycosuria data from this group. Abu-Amer et al. described a case where an SGLT2i was stopped two days before emergency bowel obstruction surgery. Despite this, persistent glycosuria was observed for a week, with EuDKA developing on POD10 [21]. It is suggested by the authors that gastrointestinal stasis affected the absorption of the drug. Additional case reports in patients with reduced gastrointestinal motility report similar findings [66, 67]. As such, we provide two conclusions. First, future studies should report glycosuria data to identify patient factors affecting SGLT2i bioavailability to refine preoperative withholding period recommendations. Second, patients with reduced gastrointestinal motility may benefit from longer SGLT2i withholding periods.

### Limitations

There are several limitations to this review. First, it relied on published case reports, which are subject to publication bias, selective reporting, and incomplete validity assessments. It is likely that this cohort of patients represents a small denomination of the already under-reported advent of adverse drug effects to regulatory agencies. It is impossible to estimate a true frequency of EuDKA and thus the results of this review may not represent the majority of cases. Second, the sample size was small, which limits the statistical power of any observations and prevents meaningful subgroup analysis. Many of the included reports provided variable and incomplete information, which limited data analysis and the ability to draw firm conclusions. Third, cases where SGLT2i-treated patients underwent emergency procedures and did not develop EuDKA were actively excluded from this narrative review, making it difficult to evaluate true risk factors. Fourth, we actively excluded patients receiving SGLT2i treatment for indications apart from T2DM, including kidney disease and heart failure, due to perceived clinical heterogeneity of reported cases. We acknowledge the risk of EuDKA is also present in such patients presenting for emergency surgery, and as the rate of prescriptions for SGLT2i continues to rise, future studies are required to investigate this association.

This is the first systematic review to investigate perioperative SGLT2i-induced EuDKA in the emergency surgery cohort specifically, and comprehensively describe the baseline demographics, clinical characteristics, perioperative considerations and outcomes in this population. Our strict eligibility criteria have allowed a more systematic dissection of our emergency surgery cohort than other reviews that have investigated EuDKA with a wider scope. Another strength is that we have conducted a literature search without language or date restriction and have attempted to exclude bias whenever possible. The trends highlighted across these cases may add to the body of work informing the perioperative understanding and management of SGLT2i-induced EuDKA in the emergency surgery population.

## Conclusion

SGLT2i-induced EuDKA in the emergency surgery context remains a diagnostic and management challenge due to its subtle biochemical profile and non-specific symptoms. This review identified potential risk factors—poor glycaemic control, obesity, and surgical stress—that were associated with more severe outcomes. Early identification, appropriate perioperative insulin and fluid management, and patient education on sick-day protocols are critical for prevention. Whilst limited by case report data, this review highlights the need for greater clinician awareness and more robust perioperative guidelines. Future prospective studies are essential to better characterise risk and guide management in this vulnerable population.

## Supplementary Information

Below is the link to the electronic supplementary material.

Supplementary file1 (DOCX 28 KB)

## Data Availability

All data generated or analysed during this study are included in this published article and its supplementary information files. The supplementary tables contain details of the search strategy, inclusion and exclusion criteria, quality appraisal checklists, and extracted case-level data used in the analysis.
