# Supplementary material for: Sodium glucose co-transporter 2 inhibitor-associated euglycaemic diabetic ketoacidosis in the emergency peri-operative period: a systematic review
Source: J Anesth. 2025 Aug 31;39(6):976–88. doi: 10.1007/s00540-025-03570-2 (PMC12647175; doi:10.1007/s00540-025-03570-2)
Supplement: Supplementary file 1 — Supplementary file1 (DOCX 28 KB) [file 540_2025_3570_MOESM1_ESM.docx]

# Supplementary Tables

| **Supplementary Table 1: Example Search Strategy** | |
| --- | --- |
| **MEDLINE and EMCARE(Ovid**) | **type 2 diabetes mellitus (T2DM) – Used Search Term Heading**  "adult-onset diabetes mellitus" OR "ketosis-resistant diabetes mellitus" OR mody OR "maturity onset diabetes" OR "maturity onset diabetes mellitus" OR niddm OR "non-insulin-dependent diabetes mellitus" OR "slow-onset diabetes mellitus" OR "stable diabetes mellitus" OR "type 2 diabetes" OR "type two diabetes"  **emergency surgery – Used Individual Key Word Search**  surger* OR perioperative OR "post operative" OR "peri-operative" OR "post-operative" OR "perioperative" or postoperative  **sglt-2 inhibitor – Used Individual Key Word Search + Search Heading**  "SGLT-2 inhibitors" OR gliflozin* OR "sglt 2 inhibitor" OR "sglt 2 inhibitors" OR "sodium glucose transporter 2 inhibitor" OR "sodium glucose transporter 2 inhibitors" OR "sodium glucose co-transporter 2 inhibitors" OR "sodium glucose cotransporter 2 inhibitors" OR 0sac974z85 OR "1-(glucopyranosyl)-4-methyl-3-(5-(4-fluorophenyl)-2-thienylmethyl)benzene - t777973" OR "6s49dgr869" OR "928672-86-0 " OR hemihydrate OR canagliflozin OR Invokana OR dapagliflozin OR “dapagliflozin propanediol monohydrate” OR empagliflozin OR ertugliflozin OR ipragliflozin OR "1 5 anhydro 1 5 4 ethoxybenzyl 2 methoxy 4 methylphenyl 1 thioglucitol" OR luseogliflozin OR "2s 3r 4r 5s 6r 2 4 chloro 3 4 ethoxybenzyl phenyl 6 methylthio tetrahydro 2h pyran 3 4 5 triol" OR sotagliflozin OR "6 4 ethylphenyl methyl 3 4 5 6 tetrahydro 6 hydroxymethyl spiro isobenzofuran 1 3h 2 2h pyran 3 4 5 triol" OR tofogliflozin OR “remogliflozin etabonate” OR “sergliflozin etabonate” OR mizagliflozin OR enavogliflozin OR atigliflozin OR bexagliflozin OR “bi 44847” OR “2 [4 chloro 3 [4 (tetrahydro 3 furyloxy)benzyl]phenyl] 6 hydroxymethyl 2 methoxytetrahydro 2h pyran 3,4,5 triol”  **euglycaemic diabetic ketoacidosis – Used Search Term Heading + Kew Word Search**  "euglycemic diabetic ketoacidosis" OR euDKA OR "diabetic ketoacidoses" OR "diabetic ketoacidosis" OR "diabetic acidoses" OR "diabetic acidosis" OR "diabetic ketoses" OR "diabetic ketosis" OR “metabolic acidosis” OR “metabolic acidosis” |

| **Supplementary Table 2: Inclusion/Exclusion Criteria** | |
| --- | --- |
| **Inclusion** | **Exclusion** |
| **Study Type:**   - Randomised Controlled Trials (RCTs) - Retrospective and Prospective Studies including cohort/case-control/case series - All languages | **Study Type:**   - Review Articles |
| **Participant Group:**   - Patients received SGLT2i therapy peri-operatively - Patients who underwent an emergency operation - Patients developing euglycemic ketoacidosis intra or postoperatively up to 30 days 🡪 defined as pH <7.30, anion gap > 10, serum glucose <13.9mmol/L, blood or urine ketone positivity   - Cases where doctor notes ‘acidosis’, but where pH is unclear are also included   - Cases where doctor ‘ketosis’ but where serum/urine ketones are unclear are also included - Patient ages of >18 - Patient history of T2DM | **Participant Group:**   - Patients who underwent elective operations - Patients newly prescribed SGLT-2 inhibitors post-operatively AND/OR not on SGLT2i prior to surgery - Patients who do not fit the euglycaemic ketoacidosis criteria - Pregnant patients |

| **Supplementary Table 3: Joanna Briggs Institute Quality Appraisal Table for Case Reports** | | | | | | | | | |
| --- | --- | --- | --- | --- | --- | --- | --- | --- | --- |
| **Article Author** | **Q1** | **Q2** | **Q3** | **Q4** | **Q5** | **Q6** | **Q7** | **Q8** | **Outcome** |
| Abu-Amer N, 2019 | Yes | Yes | Yes | Yes | Yes | Yes | Unclear | Yes | Include |
| Bteich F, 2019 | Yes | Yes | Yes | Yes | Yes | Yes | Yes | Yes | Include |
| Chandrakumar HP, 2021 | Yes | Yes | Yes | Yes | Yes | Yes | Yes | Yes | Include |
| Fisker F, 2019 | Yes | Yes | Yes | Yes | Yes | Yes | Yes | Yes | Include |
| Fustiga J, 2022 | Yes | Yes | Yes | Yes | Yes | Yes | Unclear | Yes | Include |
| Horikoshi R, 2019 | Yes | No | Yes | Yes | Yes | Yes | No | Yes | Include |
| Hung KC, 2021 | Yes | Yes | Yes | Yes | Yes | Yes | Yes | Yes | Include |
| Ito T, 2022 | Yes | Yes | Yes | Yes | Yes | Yes | No | Yes | Include |
| Jhaveri U, 2019 | Yes | Yes | Yes | Yes | Yes | Yes | No | Yes | Include |
| Kitahara C, 2021 | Yes | Yes | Yes | Yes | Yes | Yes | No | Yes | Include |
| Leon CG, 2020 | Yes | Yes | Yes | Yes | Yes | Yes | Yes | Yes | Include |
| Lindsay PJ, 2020 | Yes | Yes | Yes | Yes | Yes | Yes | No | Yes | Include |
| Ritchie DT, 2023 | Yes | Yes | Yes | Yes | Yes | Yes | No | Yes | Include |
| Takemoto Y, 2019 | Yes | Yes | Yes | Yes | Yes | Yes | Unclear | Yes | Include |
| Tsai MK, 2019 | Yes | Yes | Yes | Yes | Yes | Yes | No | Yes | Include |
| Ullah S, 2016 | Yes | Yes | Yes | Yes | Yes | Yes | No | Yes | Include |
| Wang Q, 2022 | Yes | Yes | Yes | Yes | Yes | Yes | Yes | Yes | Include |
| Wong YC, 2021 | Yes | Yes | Yes | Yes | Yes | Yes | Yes | Yes | Include |
| Yeoh HL, 2021 | Yes | Yes | Yes | Yes | Yes | Yes | Yes | Yes | Include |
| **Q1:** Were patient’s demographic characteristics clearly described?  **Q2:** Was the patient’s history clearly described and presented as a timeline?  **Q3:** Was the current clinical condition of the patient on presentation clearly described?  **Q4:** Were diagnostic tests or assessment methods and the results clearly described?  **Q5:** Was the intervention(s) or treatment procedure(s) clearly described?  **Q6:** Was the post-intervention clinical condition clearly described?  **Q7:** Were adverse events (harms) or unanticipated events identified and described?  **Q8:** Does the case report provide takeaway lessons? | | | | | | | | | |

| **Supplementary Table 4: Joanna Briggs Institute Quality Appraisal Table for Case Series** | | | | | | | | | | | |
| --- | --- | --- | --- | --- | --- | --- | --- | --- | --- | --- | --- |
| **Article Author** | **Q1** | **Q2** | **Q3** | **Q4** | **Q5** | **Q6** | **Q7** | **Q8** | **Q9** | **Q10** | **Outcome** |
| Mehta PB, 2022 | Yes | Yes | Yes | Yes | Yes | Yes | Yes | Yes | Yes | Yes | Include |
| Wang R, 2021 | Yes | Yes | Yes | Yes | Yes | Yes | Yes | Yes | Yes | Yes | Include |
| **Q1:** Were there clear criteria for inclusion in the case series?  **Q2:** Was the condition measured in a standard, reliable way for all participants included in the case series?  **Q3:** Were valid methods used for identification of the condition for all participants included in the case series?  **Q4:** Did the case series have consecutive inclusion of participants?  **Q5:** Did the case series have complete inclusion of participants?  **Q6:** Was there clear reporting of the demographics of the participants in the study?  **Q7:** Was there clear reporting of clinical information of the participants?  **Q8:** Were the outcomes or follow up results of cases clearly reported?  **Q9:** Was there clear reporting of the presenting site(s)/clinic(s) demographic information?  **Q10:** Was statistical analysis appropriate? | | | | | | | | | | | |

| **Supplementary Table 5: Types of Surgery in reported cases** | | |
| --- | --- | --- |
| **Location of Surgery** | **Type of Surgery** | **No. of Reported Cases** |
| Gastrointestinal | Laparoscopic gastric wedge resection for gastric perforation | 1 |
|  | Laparoscopic cholecystectomy | 2 |
|  | Emergency laparotomy after laparoscopic distal pancreatectomy | 1 |
|  | Bowel resection | 1 |
| Cardiovascular | CABG | 4 |
|  | Catheter-directed thrombolysis of popliteal graft | 1 |
|  | Biventricular ICD placement | 1 |
| Orthopaedic | ORIF for femoral neck fracture | 1 |
|  | ORIF for femoral shaft fracture | 1 |
|  | ORIF for tibia fracture and spinal fusion | 1 |
|  | ORIF for humerus fracture | 1 |
|  | ORIF for ulna fracture and pedicle screw fixation of spinal fractures | 1 |
|  | Laminectomy | 2 |
|  | Total hip replacement | 1 |
| Neurological | VP Shunt Exchange | 1 |
|  | Cerebral angiography | 1 |
|  | Craniotomy | 1 |
| Urological and Gynaecological | Total hysterectomy | 1 |
|  | Cystoscopy | 1 |
| Soft Tissue | Debridement | 2 |
|  | Incision and Drainage | 2 |
| Other | Thoracoscopic debridement and intrathoracic lavage | 1 |
|  | Trabeculectomy for Acute Angle Glaucoma | 1 |
| **Total** |  | **30** |
| *CABG* Coronary Artery Bypass Graft, *ICD* Implantable Cardioverter-Defibrillator, *ORIF* Open Reduction Internal Fixation, *VP* Ventriculoperitoneal | | |
